# Novel diagnostic techniques in interstitial lung disease

**DOI:** 10.3389/fmed.2023.1174443

**Published:** 2023-04-28

**Authors:** Laura M. Glenn, Lauren K. Troy, Tamera J. Corte

**Affiliations:** ^1^Department of Respiratory and Sleep Medicine, Royal Prince Alfred Hospital, Camperdown, NSW, Australia; ^2^Central Clinical School, The University of Sydney School of Medicine, Sydney, NSW, Australia; ^3^NHMRC Centre of Research Excellence in Pulmonary Fibrosis, Camperdown, NSW, Australia

**Keywords:** interstitial lung disease, diagnostic techniques, biomarker, genomics, machine learning, optical coherence tomography, electronic nose

## Abstract

Research into novel diagnostic techniques and targeted therapeutics in interstitial lung disease (ILD) is moving the field toward increased precision and improved patient outcomes. An array of molecular techniques, machine learning approaches and other innovative methods including electronic nose technology and endobronchial optical coherence tomography are promising tools with potential to increase diagnostic accuracy. This review provides a comprehensive overview of the current evidence regarding evolving diagnostic methods in ILD and to consider their future role in routine clinical care.

## Introduction

Interstitial lung diseases (ILDs), despite being a large group of diverse disorders, are grouped together since they are invariably characterized by inflammation and/or fibrosis of the lung parenchyma (1). Affected patients often have similar symptoms including cough and breathlessness. While some ILDs share overlapping radiological and histopathological features, there is a wide range of natural histories and responses to treatment across the ILD spectrum. Delays in diagnosis can lead to missed opportunities to intervene early and potentially prevent progressive pulmonary fibrosis (PPF), a recently defined disease behavior entity which is associated with high morbidity and mortality (1, 2). Idiopathic pulmonary fibrosis (IPF), the most common ILD, is universally progressive; however, antifibrotic therapy with nintedanib or pirfenidone significantly reduces the rate of decline in lung function in patients with both early and moderate disease. A similar treatment effect with nintedanib has been demonstrated in other causes of PPF (3).

Unfortunately, a specific ILD diagnosis remains elusive in up to 20% of cases; and is often delayed (4). The reasons for this are multifactorial, including heterogeneous clinical presentation, radiological and pathological features even within diagnostic subgroups, the rarity of some ILDs, under-recognition of ILD features in the primary care setting; and importantly, global inequality of access to expert care (1, 5–10). The ILD multidisciplinary meeting (MDM) is the current recommended “gold standard” method for diagnosing ILDs, yet several studies have demonstrated suboptimal agreement between clinicians and ILD MDMs for individual diagnoses (11–18).

While this might initially seem discouraging, the future is promising. Recent research into novel diagnostic techniques and targeted therapeutics in ILD is moving the field toward increased precision and improved patient outcomes (19).

## Conventional and emerging ILD diagnostic tools in use

Detailed clinical assessment through history-taking and examination, has always been important in ILD diagnosis and remains an essential first step in the modern systematic approach (1, 20). A history of exposures in the home and workplace should be obtained, including to mold, asbestos, and other relevant occupational dusts, which may point toward an underlying inciting agent. Recently, the use of a standardized questionnaire has been shown to increase both diagnostic confidence and antigen recognition in chronic hypersensitivity pneumonitis (21). Similarly, physical signs detected during the examination might support presence of an ILD (nail clubbing, fine inspiratory crackles) or suggest an underlying connective tissue disease diagnosis (sclerodactyly, inflammatory arthritis, typical skin rash).

Serological tests for autoantibodies may also point toward systemic autoimmune disease. Many ILD centers will screen for antinuclear antibodies, rheumatoid factor as well as more specific connective tissue disease-associated antibodies such as anti-double stranded DNA, anti-Ro and anti-La, anti-Scl-70, anti-ribonucleoprotein, anti-cyclic citrullinated peptide (CCP) and the anti-tRNA synthetase antibodies. New immunoassay platforms and an extended panel of highly specific myositis antibodies (including antibodies to PM-Scl, MDA-5, Mi2, Ku, TIF1γ, and NXP2) are now also part of routine testing in many expert centers worldwide. Similarly, nailfold capillaroscopy has recently been suggested as an adjunctive clinical test to assess for the presence of vascular changes associated with systemic sclerosis and other connective tissue diseases such as the idiopathic inflammatory myopathies and mixed connective tissue disease (22–24). The high specificity of abnormal nailfold capillaroscopy findings has rendered it an integral tool in the diagnosis of these diseases. Although generally performed using conventional microscopy, nailfold capillaroscopy has also been performed at the bedside using smartphone-dermatoscopy (23).

A critical technological advance was the development of computed tomography (CT) in the 1970s, enabling detailed axial imaging of the lung parenchyma for the first time (20). The Fleischner Society and international expert groups have subsequently published numerous glossaries, white papers and clinical practice guidelines describing classifications of interstitial lung disease patterns on high resolution CT (HRCT) chest imaging, including usual interstitial pneumonia (UIP), non-specific interstitial pneumonia (NSIP) and other patterns which are associated with specific ILDs (25–32). In over two-thirds of ILD cases, the synthesis of HRCT pattern and clinical information enables a diagnosis to be made at the ILD MDM (1).

For patients in whom clinical and HRCT findings are not sufficiently characteristic to allow confident diagnosis, further investigation including bronchoscopy or tissue sampling for histopathological classification may be required. Flexible bronchoscopic diagnostic techniques including bronchoalveolar lavage (BAL) and transbronchial biopsy were integrated into clinical practice around the same time as early CT imaging (20). BAL remains a routinely performed diagnostic test in patients with newly detected ILD who do not have a definite UIP pattern on HRCT. Cellular analysis of BAL fluid can reveal lymphocytosis helpful for distinguishing hypersensitivity pneumonitis from IPF or sarcoidosis; or high eosinophil counts suggesting a diagnosis of eosinophilic pneumonia. BAL cultures can help exclude infection as an alternate cause of lung infiltrates prior to institution of specific immunosuppressive or antifibrotic therapies. Transbronchial biopsies with forceps (TBB) are not routinely recommended for histopathological diagnosis due to having generally low diagnostic yield and moderate risk of pneumothorax (31). However, they might be considered on a case-by-case basis for diagnosis of airway-centered processes such as sarcoidosis or organizing pneumonia, where the sensitivity is much higher. There is still widespread use for these indications in centers where clinicians have expertise in TBB.

Historically, tissue sampling required surgical lung biopsy (via open thoracotomy in the 1950s and 1960s and via video-assisted thoracoscopic surgery (VATS) in more recent decades) if the patient was considered suitable for thoracic surgery (20). Within the last decade, transbronchial lung cryobiopsy (TBLC) has emerged as a less invasive tissue sampling procedure for ILD diagnosis, with good diagnostic yield of ~80% (33–35), and comparable accuracy to VATS biopsy in some studies when considered within the MDM (36). Although the quality of available evidence is low, current systematic review and meta-analysis data demonstrate TBLC to have a lower 30-day mortality rate than VATS (0.6% versus 1.7%), and low risk of severe complications such as prolonged air leak or acute ILD exacerbation. These factors make TBLC a favorable alternative to SLB for tissue sampling in centers with expertise, with uptake of the procedure by centers across the world (33).

These diagnostic test results are considered by a multidisciplinary team within the MDM to formulate an ILD diagnosis – the recommended approach in IPF clinical practice guidelines in the last decade (2, 31). In recent years, there has been divergence of opinion regarding the need for strict pursual of specific ILD diagnosis versus “lumping” patients based on their expected and observed disease behaviors including those with a progressive fibrotic phenotype and those with non-progressive disease (37–39). Indeed, this is reflected in the variable recommendations of different MDMs. While some MDMs will recommend referral for biopsy in the event of unclassifiable or low confidence diagnoses, others will generate a working diagnosis (of lower confidence) to facilitate treatment institution. This is frequently related to a patient’s current degree of disability and burden of comorbidities, limiting ability to proceed to biopsy and therefore attainment of a high confidence diagnosis. In the future, standardization of the ILD MDM, including approach to clinical decision-making, will be required to reduce heterogeneity of outputs. Indeed, although the MDM is considered the current “gold standard” method for ILD diagnosis, there is a clear need for simpler, non-invasive, and more precise ILD diagnostic tests.

## Emerging ILD diagnostic tests under investigation

### Genetic testing

The advent of biobanks and evolution of methods for molecular analysis, including targeted next-generation sequencing and whole genome sequencing in the early 2000s revolutionized the concept of genetic testing in ILD (40–42). Single nucleotide polymorphisms (SNPs) in genes encoding for proteins expressed by airway epithelial cells, such as *MUC5B*, have been identified to have both diagnostic and prognostic significance in IPF and other fibrotic ILDs such as rheumatoid arthritis-associated ILD and chronic hypersensitivity pneumonitis. Patients with at least one *MUC5B* risk allele have a more than threefold increased risk of developing pulmonary fibrosis (41). However, IPF patients with at least one of these alleles have been shown to have slower disease progression and improved survival compared to patients without a risk allele in retrospective and post-hoc analyses (41). The first whole genome sequencing study of 2,180 IPF cases, recently published, found single rare variants in *TERT* and *RTEL1* genes to be significantly associated with IPF development, and confirmed previously studied association with other more common genetic variants. SNP-heritability in IPF was estimated to be 32% (43).

Importantly, mutations in telomere-related genes such as *TERT* and *PARN* confer substantial risk of familial pulmonary fibrosis and are associated with more rapidly progressive disease, as well as poorer outcomes with immunosuppressive therapy and with lung transplantation (40, 44). A recent genome-wide meta-analysis of IPF patients showed a variant in the RNA antisense gene of protein kinase N2 (PKN2), rs115982800, to be significantly associated with FVC decline. Interestingly, no other genetic variants were associated with lung function decline, however this may have been due to study underpowering (45). Variability in the association between specific genetic variants and IPF increasingly suggest that it is a more heterogenous disease than traditionally thought, with different underlying pathophysiological mechanisms (46, 47).

Genetic variants that have been associated with ILD are described in Table 1 (40–47). In addition to direct cellular injury mediated by these genetic variants, pulmonary fibrosis may also be a consequence of chronic lung inflammation due to genetic mutations causing systemic autoimmune disease; for example, CTLA-4 haploinsufficiency with autoimmune infiltration (CHAI syndrome), or chronic respiratory infections; for example, hyper-IgE syndrome.

**Table 1:** Gene polymorphisms associated with ILD.

Gene(s)	Phenotype
MUC5B	Risk allele (rs35705950) associated with increased susceptibility to familial pulmonary fibrosis (heterozygous OR 6.8, 95% CI 3.9–12; homozygous OR 20.8, 95% CI 3.8–113.7), IPF (heterozygous OR 9.0, 95% CI 6.2–13.1; homozygous OR 21.8, 95% CI 5.1–93.5). Also associated with UIP-pattern ILD in rheumatoid arthritis, and hypersensitivity pneumonitis patients.
Surfactant-related genes (SFTPC, SFTPA2, SFTPA1)	Heterozygous mutations associated with familial pulmonary fibrosis, RA-ILD
Other genes associated with protein expression and function	AKAP13, ATP11A, DPP9, FAM13A, DSP, OBFC1 variants associated with increased susceptibility to IPF and other idiopathic interstitial pneumonias in genome-wide association studies
Telomere-related genes (TERT, TERC, PARN, RTEL1, NAF1, DKC1, TINF2)	Mutations identified in up to 1/4 of familial pulmonary fibrosis patients and 1/10 sporadic IPF patients. Up to ½ patients with heterozygous variant will have non-IPF diagnosis (CTD-ILD, HP, RA-ILD); however, prognosis is similar to patients with UIP. Fibrosis of other organs, including liver cirrhosis and myelofibrosis, premature hair graying and/or other features of a “short telomere syndrome” may also be seen.

Variable penetrance of ILD-associated alleles, limited data on specific treatment response and limited access to genetic counseling and testing outside of tertiary centers currently limit the widespread implementation of genetic testing. However, it is increasingly used within the ILD multidimensional diagnostic paradigm to inform risk of progressive fibrotic ILD and treatment response for affected individuals with suspected familial pulmonary fibrosis and their family members (40). Genetic testing should also be considered where there is suspicion for a “short telomere syndrome,” and evidence is mounting that screening of unaffected family members may be reasonable to facilitate earlier diagnosis and treatment institution.

#### Key points

1. Genetic variants with diagnostic and prognostic importance have been identified in familial pulmonary fibrosis and other ILDs, including single nucleotide polymorphisms in telomere-related genes, surfactant-related genes and other genes associated with protein expression and function.
2. Where access is available, genetic testing should be considered for all patients with a family history of ILD or who have features of a telomeropathy.

### Other molecular testing, including serum biomarkers

RNA sequencing and genomic classifier testing represent novel diagnostic methods of significant interest (48). RNA sequencing involves the use of high throughput sequencing technologies to identify and quantify RNA transcripts in either whole tissue or single cells (49). Analysis of the transcriptome can identify differentially expressed or regulated genes between diseased tissues compared with healthy tissues, with the aim of delineating biological mechanisms or pathways underlying disease pathogenesis. Multiple studies have evaluated the use of both bulk RNA sequencing and single-cell RNA-sequencing (scRNA-seq) to analyze fibrotic lung tissue samples (50).

A genomic classifier, developed from RNA sequencing data, has been proposed as a novel diagnostic tool that might increase diagnostic yield and accuracy in ILD. The Envisia™ genomic classifier, which employs a machine learning algorithm developed to classify UIP versus non-UIP histopathological pattern ILDs, uses bulk RNA sequencing data obtained from high throughput sequencing of exome-enriched RNA extracted from transbronchial lung biopsies or transbronchial lung cryobiopsies (TBLCs). Several subsequent studies have demonstrated the classifier to have high specificity (≥86%) to predict UIP, however its sensitivity was as low as 68% (51–54). When results were presented within the ILD multidisciplinary meeting, the genomic classifier increased diagnostic confidence for patients with probable UIP (55). However, the classifier had less impact on the proportion of high confidence diagnoses than the TBLC result; and the 2022 ATS/ERS/JRS/ALAT IPF clinical practice guideline update has made no recommendation for or against the use of genomic classifier testing in fibrotic ILD diagnosis (2).

**Figure 1:**
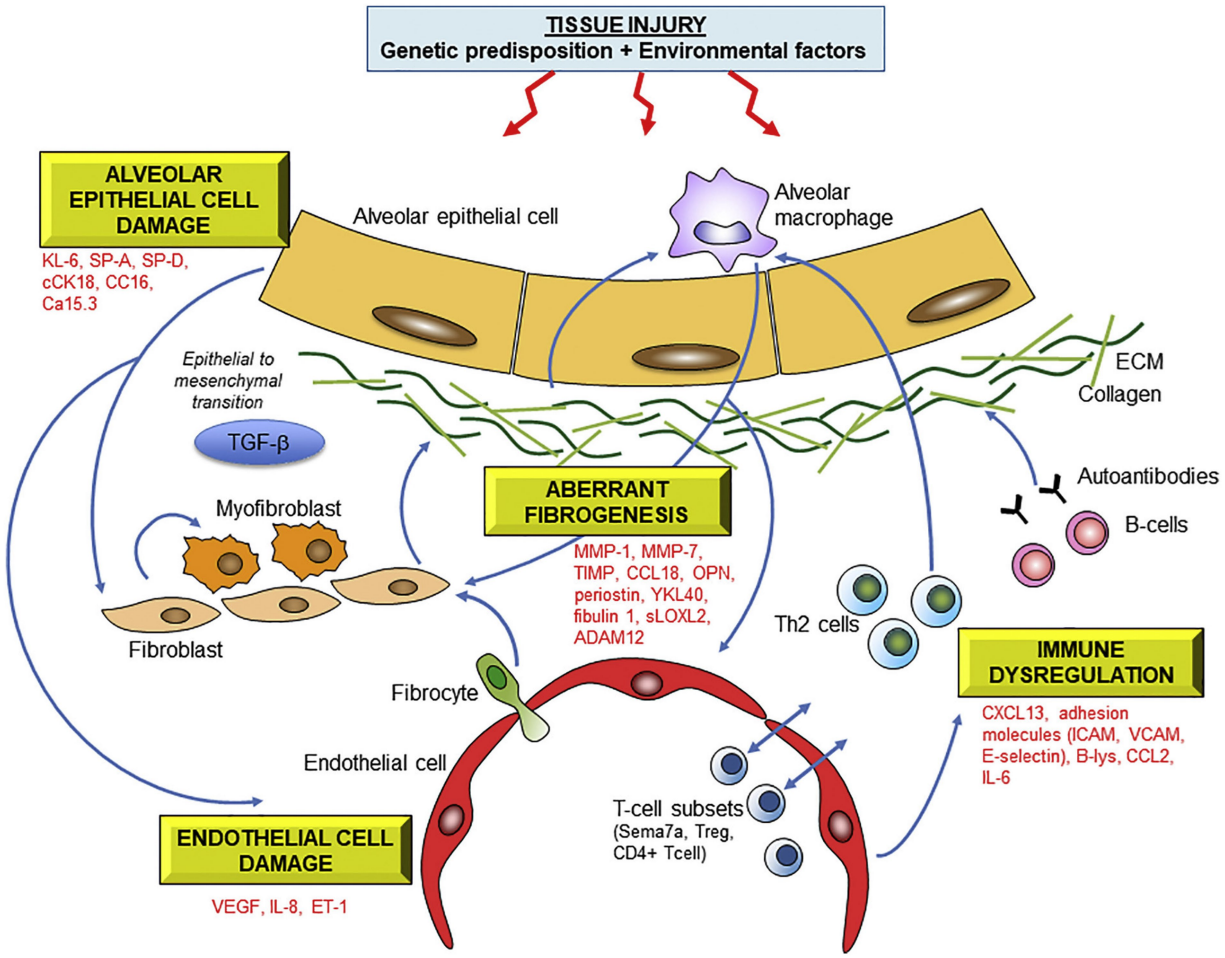
Molecular mechanisms underlying fibrotic lung injury and potential serum biomarkers of ILD. Abbreviations: KL-6 Krebs von lungen 6; SP-A/SP-D surfactant protein A/D; cCK18 cleaved cytokeratin 18; CC16 Clara cell protein 16; MMP matrix metalloproteinase; OPN osteopontin, HSP47 heat-shock protein 47; sLOXL2 serum lysyl oxidase-like 2; ADAM a disintegrin and metalloprotease; CCL18 chemokine ligand 18; ICAM intercellular adhesion molecule; VCAM vascular cell adhesion molecule; CXCL13 C-X-C motif chemokine 13; CCL2 CC chemokine 2; IL-6 interleukin-6. Reprinted from Pharmacology and Therapeutics, 202, Jee et al. (56), with permission from Elsevier.

The quest for a biomarker signature from peripheral blood to improve specific ILD diagnosis and accurate prediction of prognosis at first presentation has been world-wide. Multiple biomarkers have been identified and several have been validated across multiple cohorts. Peripheral blood biomarkers with promise for future translation to clinical use include matrix metalloproteinase 7 (MMP-7), Krebs von den Lungen (KL-6), osteopontin (OPN), periostin and surfactant protein D (SP-D); and various other cytokines, chemokines, growth factors, matrix metalloproteinases, extracellular matrix proteins and markers of epithelial injury and apoptosis (56) (Figure 1).

The landmark PROFILE study of incident cases of fibrotic interstitial lung disease involved a discovery analysis of concentrations of 123 previously described serum biomarkers using multiplex immunoassay, followed by a validation analysis employing independent immunoassays for each of the three identified biomarkers. SP-D, carbohydrate antigen 19.9 (CA19-9) and cancer antigen 125 (CA-125) were identified as prognostically important biomarkers, with higher baseline values of SP-D and CA 19–9 significantly associated with disease progression; and increasing levels of CA-125 over a three-month time period were associated with higher mortality risk (57). More recently, a panel of serum biomarkers was assessed in three separate IPF cohorts. Osteopontin, MMP-7, intercellular adhesion molecule-1 (ICAM-1) and periostin were differentially expressed between progressive and stable IPF. The investigators developed a statistical model incorporating these four biomarkers which was able to predict risk of disease progression in each cohort (58). Systematic review and meta-analysis data confirms the association between baseline MMP-7 levels and outcomes in untreated IPF, including disease progression and mortality risk (59).

Proteomic analysis of blood biomarkers for prediction of disease behavior holds promise as a future tool with potentially increased precision over individual biomarker measurement. Analysis of a cohort of connective tissue disease-associated ILD, chronic hypersensitivity pneumonitis and unclassifiable ILD patients’ serum samples at diagnosis identified and validated 17 novel biomarkers associated with progressive pulmonary fibrosis, including CXCL17 (C-X-C motif chemokine ligand 17) and TGFA (transforming growth factor alpha) (60). A proteomic “signature” of PPF was then developed and validated using machine learning algorithms, incorporating 12 serum biomarkers, which had a sensitivity of 0.90 for identifying a progressive fibrosing ILD phenotype. Patients with a high-risk proteomic signature experienced significant deterioration in their forced vital capacity (FVC) of −227.1 mL (95% CI −286.7 mL to −167.5 mL); as opposed to patients with low-risk proteomic signature whose FVC did not decline over 12 months (60).

Liquid biopsy is another emerging molecular diagnostic technique, which involves the extraction and analysis of circulating cell-free (ccf) DNA fragments from blood. Levels of ccfDNA have been shown to be significantly increased in IPF patients compared with age- and sex-matched healthy volunteers, and were also significantly associated with disease severity as measured by the previously validated IPF GAP (gender-age-physiology) score. In that study, the median plasma expression of ccfDNA fragments, 104 ng/mL differentiated between cases of more advanced IPF (GAP score 2–3), versus more mild disease (GAP score 1) (61). Interestingly, discordance between ccfDNA and genomic DNA extracted from peripheral blood mononuclear cells in some IPF patients, but not healthy controls, was observed. It has been hypothesized that ccfDNA in peripheral blood might encode genetic information present in the diseased lungs which is not measurable in genomic DNA (61).

Bulk RNA sequencing analyses the average expression level of genes across all cells in a tissue, as opposed to single-cell RNA sequencing, which quantifies differential gene expression by specific cell populations within tissues. Bulk RNA sequencing studies have contributed to increased knowledge of the pathobiology of IPF and other fibrotic lung diseases (62–64). Bulk RNA extracted from IPF lung explants demonstrated the severe IPF transcriptome to be enriched in pathways of T-cell infiltration and activation and tumor development, and a specific subset of genes correlated with patients’ forced vital capacities (FVC) (65). A 2019 exploratory study analyzed bulk RNA from IPF explants, and then subsequently performed micro-CT scanning and standard immunohistochemistry on cores of tissue taken from differentially affected lung regions (66). A core set of differentially expressed genes was identified to be present in the IPF lung before fibrosis was even histologically evident, and their profile was further altered in areas of more advanced fibrosis.

Single-cell RNA-sequencing studies, largely performed using explanted IPF lungs in comparison with control healthy donor lungs, have identified numerous upregulated and downregulated genes in fibrotic lungs expressed by specific cell populations (67). For example, altered alveolar type II epithelial cell and mesenchymal cell gene expression has been associated with the activation and proliferation of fibroblasts and excessive extracellular matrix deposition seen in IPF (50). Techniques such as confocal microscopy, immunohistochemistry, in-situ hybridization and/or proteomic analysis of lung tissues, BAL fluid and/or serum have been employed concurrently to ascertain the functional correlation of altered gene expression (68). For example, collagen-producing cell subpopulations in fibrotic lungs were observed to be concentrated within fibroblastic foci in fibrotic lungs (69). Table 2 summaries insights into the fibrotic lung transcriptome gained from single cell RNA sequencing studies performed in IPF and other fibrotic ILDs.

**Table 2:** Outcomes of single cell RNA sequencing studies in fibrotic ILD.

Study	Year	Cell population studied	Insights into human fibrotic lung transcriptome
Nemeth et al. (50)	2020	Alveolar type II epithelial (AE2) cells, mesenchymal cells	Activation and proliferation of fibroblasts, excessive extracellular matrix deposition in IPF
Tsukui et al. (69)	2020	Fibroblasts	CTHRC1-expressing fibroblasts demonstrated in fibroblastic foci in fibrotic lungs from IPF and systemic sclerosis patients
Xu et al. (70)	2016	Alveolar epithelial cells	Frequent co-expression of multiple surface markers associated with indeterminate differentiation and aberrant activation of downstream signaling pathways, for example TGF-β, in IPF
Xi et al. (71)	2017	AE2 cells	Subpopulation of AE2 cells with HIF1α-driven impaired differentiation and migration in IPF
Morse et al. (72)	2019	Alveolar epithelial cells, macrophages	Increased fibroblasts and basal, ciliated, goblet and club cells in IPF lower lobes compared to healthy lungs; decreased alveolar epithelial cells and marked alterations in inflammatory cell populations, including discrete macrophage subsets highly expressing SPP1 and MERTK
Reyfman et al. (73)	2019	Alveolar epithelial cells, macrophages	Heterogeneity in epithelial cell and alveolar macrophage gene expression, and populations of profibrotic alveolar macrophages in fibrotic lungs
Valenzi et al. (74)	2019	Myofibroblasts	Upregulated expression of collagen and other profibrotic genes by myofibroblasts in systemic sclerosis-associated ILD lungs
Adams et al. (75)	2020	Epithelial and stromal cells, macrophages, vascular endothelial cells	Identified a population of aberrant cells at edge of fibroblast foci that co-express basal epithelial, mesenchymal, senescence and developmental markers; profibrotic macrophage populations in IPF lungs
Carraro et al. (76)	2020	Epithelial cells	Restriction of basal-to-ciliated differentiation in IPF
Habermann et al. (77)	2020	Epithelial cells	Extracellular-matrix producing epithelial cell population highly enriched in IPF lungs
Liu et al. (78)	2020	Fibroblasts	Altered gene expression in fibroblast populations from age-matched fibrotic lungs from mice and humans
Mayr et al. (79)	2021	Alveolar epithelial cells	Altered alveolar epithelial cell expression of CRTAC1, which encodes a glycosylated extracellular matrix protein; also reflected in bronchoalveolar lavage fluid and patient plasma

AE2, alveolar epithelial type 2 cells; IPF, idiopathic pulmonary fibrosis; CTHRC1, collagen triple helix repeat containing-1; TGF-β, transforming growth factor beta; HIF1α, hypoxia inducible factor 1 subunit alpha; SPP1, secreted phosphoprotein 1; MERTK, tyrosine-protein kinase Mer; ILD, interstitial lung disease; CRTAC1, cartilage acidic protein 1.

Clearly, RNA sequencing (RNA-seq) has advanced knowledge of the molecular and pathway alterations underlying fibrotic lung diseases. Additionally, RNA-seq and these other molecular tests have potential for future use in identification of novel biomarkers enabling “smart splitting” of ILD patients at diagnosis based on respective components of inflammation and fibrosis in their disease pathogenesis. In addition, this may lead to identification of more specific drug targets and improved disease behavior prediction.

#### Key points

1. RNA sequencing, involving the use of high throughput sequencing technologies to identify and quantify RNA transcripts in whole tissues or single cells, has identified differentially expressed genes between lung tissue obtained from ILD patients and healthy controls.
2. A genomic classifier developed from RNA sequencing data has demonstrated high specificity in differentiating UIP from non-UIP pattern ILDs, yet it has low sensitivity and little additional impact on diagnostic confidence at ILD MDM.
3. Peripheral blood biomarkers, including MMP-7 and SP-D, have been demonstrated to differentiate between specific ILD subtypes; and also predict disease behavior and response to treatment. These biomarkers need to be assessed for their utility in the integrated clinical setting.
4. Additional novel molecular diagnostic techniques include proteomic and metabolomic analysis of blood, bronchoalveolar lavage specimens and lung tissue samples; and “liquid biopsy” (analysis of cell-free circulating DNA fragments in blood). These techniques hold significant promise for improving future precision disease profiling and guiding therapeutic decision-making, yet require more extensive study before they are ready for implementation into routine clinical care.

### Artificial intelligence technologies: deep-learning based radiologic and histopathological assessment

Computer-based deep learning techniques, consisting of convolutional neural network (CNN) algorithms able to autonomously detect features in images, have the potential to revolutionize ILD assessment by reducing human inter-observer variability in interpretation of diagnostic tests (80).

A 2021 systematic review, including 19 retrospective studies, demonstrated deep learning-based assessment of ILD CT scans to have good diagnostic accuracy for classification of ILD pattern, between 76.4–95.1%, when considering consensus radiologist assessment as the reference standard (81).

Walsh et al. (82) conducted a case-cohort study for deep learning algorithm development and assessment and included 1,157 high resolution CT scans obtained from patients with fibrotic ILD, separated into training, validation, and testing cohorts. An initial CNN segmented the lungs and then resampled them to create a maximum of 500 four-slice combinations per scan, creating montages which were fed into the training dataset. The final algorithm was able to classify each HRCT using the 2011 ATS/ERS/JRS/ALAT IPF guidelines with a diagnostic accuracy of 76.4% on the first testing set, and 73.3% on the second testing set which was comparable to the median accuracy of thoracic radiologists (70.7%) (82). In view of its ability to provide rapid and reproducible results, the investigators concluded that this technology could be beneficial for ILD assessment in centers without access to radiologists with ILD expertise. More recently, a retrospective multicenter study assessed the diagnostic ability of a deep-learning algorithm when applied to 1,239 high resolution CT scans of fibrotic ILD. The algorithm performed superiorly to two expert radiologists in predicting histopathologic UIP (area under the receiver-operating characteristic curve, 0.87 vs. 0.80, *p* < 0.05) (83).

**Figure 2:**
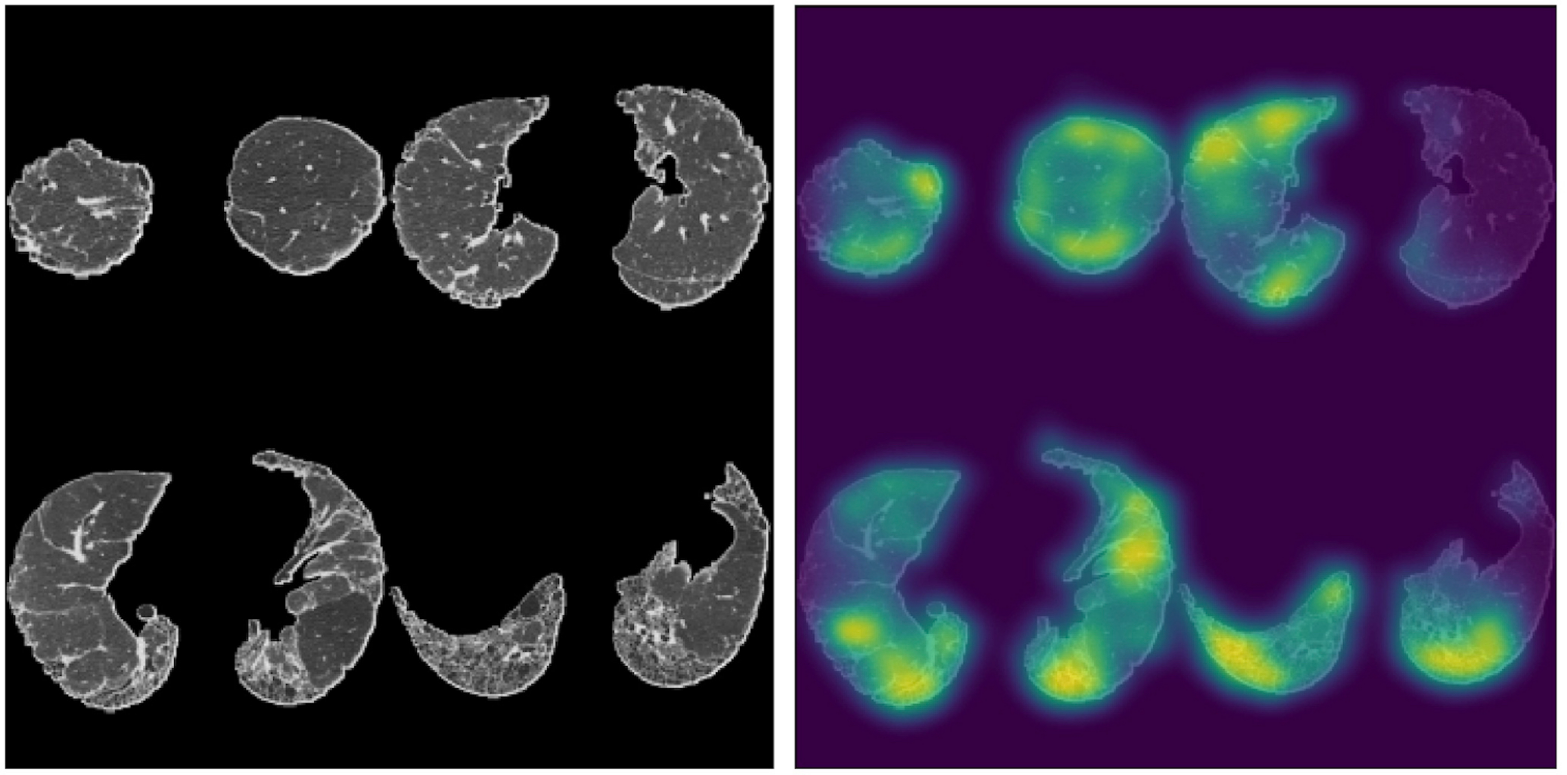
Four slice montage generated from a patient with an assigned HRCT diagnosis of probable UIP based on two expert thoracic radiologists, accompanied by a saliency map depicting parts of the lungs most influential in SOFIA-based decision-making. SOFIA probabilities for this montage were UIP: 0.224, probable UIP: 0.764, indeterminate for UIP 0.012, alternative diagnosis: 0.000. The SOFIA deep-learning algorithm generates up to 500 four axial slice montages from an HRCT scan by segmenting the lungs into quarters (excluding the apical 10%) and then randomly selecting slices from each quarter. The algorithm generates a set of numbers from 0 to 1, totalling 1.0, representing the probability of each of the UIP diagnosis categories (definite UIP, probable UIP, indeterminate, alternative diagnosis); which is the average probability for each diagnostic category across all the montages generated for each HRCT (85). Sourced and reproduced with permission from Simon L. F. Walsh, M.D., F.F.R.R.C.S.I., National Heart and Lung Institute, Imperical College London, London, United Kingdom.

Larger recent studies of HRCT scans obtained from participants in the Australian IPF Registry have also assessed the prognostic ability of deep learning algorithms. The extent of lung fibrosis on baseline HRCT, as assessed by data-driven texture analysis, a deep learning technique, significantly correlated with annual rate of decline in forced vital capacity and diffusing capacity for carbon monoxide (84). Another study employing the Systematic Objective Fibrotic Imaging Analysis Algorithm (SOFIA), which was also developed and validated in the identification of UIP-like features on HRCT in patients enrolled in the Australian IPF registry, demonstrated deep learning-based radiologic UIP probability to be predictive of survival in multivariable analysis, where radiologist-determined UIP probability was not (85). Figure 2 demonstrates SOFIA analysis of an HRCT montage.

The ability for deep learning algorithms to be applied in assessment of ILD histopathology specimens also requires consideration. Pilot studies have assessed automated digital quantification of extent of fibrosis in digital images of whole lung sections (86). One study of 71 IPF patients enrolled in the Finnish IPF registry developed and then tested the ability of a semi-supervised deep learning algorithm to identify and quantify specific ILD features in lung tissue samples. The most representative hematoxylin and eosin-stained slide for each patient was scanned at 40× magnification and 20 of the resulting whole slide images were used to train the algorithm. An expert pathologist manually annotated pathognomonic features in the images to train the model. In this cohort, increased number of fibroblastic foci were significantly associated with shortened survival; and high percentages of interstitial and intra-alveolar inflammatory cells were associated with prolonged survival (87).

#### Key points

1. Computer-based algorithmic analysis of radiology and histopathology in ILD, using deep-learning techniques, may be able to improve diagnostic and prognostic precision by reducing human inter-observer variability in their interpretation.
2. Deep-learning based assessment of radiologic UIP probability has been demonstrated to have excellent prognostic utility for mortality, and this technology may be integrated into ILD care, particularly in centers without access to expert radiologists (or pathologists).

### Endobronchial optical coherence tomography

Optical coherence tomography (OCT) is a non-ionizing imaging technology that employs low coherence light waves to capture high resolution of soft tissues to a resolution of <10 μm. Endogenous tissue serves as an optical scattering media, enabling measurement of the time delay and magnitude of backscattered light to generate cross-sectional images (88, 89).

Endobronchial OCT (EB-OCT) is performed by passing an OCT probe through the working channel of a flexible bronchoscope to evaluate peripheral and subpleural lung tissue in-vivo. By conducting helical scanning and subsequent pullback of the probe, a three-dimensional reconstruction of sequential images along the airway path is produced. Additional advantages include the ability to image large tissue volumes to microscopic resolution without the need for tissue removal, as in surgical lung biopsy or transbronchial lung cryobiopsy, both associated with significantly higher risk of patient morbidity and mortality than diagnostic bronchoscopy (88).

Like exhaled breath analysis and other novel ILD diagnostic techniques, studies investigating the application of endobronchial OCT in ILD are scarce. Historically, this technology has been used for assessment of asthma and other airways diseases, for example, to assess airway caliber and extent of airway remodeling and quantify airway mucous (90–93). It has also been employed during bronchoscopic interventions for real-time imaging during treatment of airway obstruction (94, 95), and to guide lymph node or peripheral pulmonary nodule sampling via transbronchial needle aspiration for lung cancer diagnosis (96). More recently, small studies have demonstrated utility of EB-OCT in ILD diagnosis for patients with non-diagnostic HRCT or indeterminate biopsy. For example, by detection of microscopic honeycombing not seen on HRCT or by distinguishing between traction bronchiectasis or bronchiolectasis and microscopic honeycombing in the setting of a false-positive radiologic UIP diagnosis (97).

A 2021 prospective diagnostic accuracy study comparing EB-OCT to surgical lung biopsy in 27 patients with unclassifiable or low confidence fibrotic ILD diagnoses, demonstrated EB-OCT to have a sensitivity of 100% (95% CI 75.8–100%) and specificity of 100% (95% CI 79.6–100%) for both histopathologic diagnosis of UIP, as assessed by expert pathologist assessment of surgical lung biopsy specimens; and clinical diagnosis of IPF, as determined by the treating respiratory physician (88). Importantly, high agreement was also demonstrated between EB-OCT (interpreted by both an EB-OCT expert pathologist and novice pathologists who were trained in EB-OCT interpretation during the study) and histopathologic ILD pattern (weighted k:0.87 [0.72–1.0]). EB-OCT criteria used to distinguish between ILD diagnoses were developed from review of previous studies in IPF, lung cancer and other pulmonary pathologies. Features included subpleural fibrosis replacing normal alveolar tissue and microscopic honeycombing in UIP, non-destructive interstitial fibrosis in NSIP and fibrosis around airways with preserved distal alveolar architecture in “airway-centered fibrosis” (88).

These results are certainly very promising and propose EB-OCT as both a novel diagnostic tool and for use in monitoring treatment response and/or disease progression through serial quantifications of extent of lung fibrosis. Yet, larger studies in multiple sites are needed before EB-OCT is ready for implementation in routine clinical practice. It will be important to evaluate inter-observer variability in EB-OCT interpretation; particularly since poor inter-clinician (and inter-meeting) agreement are problems frequently encountered in traditional histopathologic ILD diagnosis by surgical lung biopsy and in the current “gold standard” method, the ILD multidisciplinary meeting (98). Technical issues such as validated methods for confirmation of correct subpleural EB-OCT probe positioning will also require further consideration (99). The use of polarization-sensitive EB-OCT, which has the added ability to detect birefringence from collagen in fibrosis, has recently been investigated as a tool for quantification of fibrosis in ILD; with positive early results (100).

#### Key points

1. Endobronchial OCT is a non-invasive, non-ionizing radiation technology which uses low coherence light waves to generate high resolution images of soft tissues.
2. Preliminary diagnostic accuracy studies suggest OCT is a promising method with high sensitivity and specificity for identifying UIP, and for differentiating between ILD subtypes through assessment of features such as subpleural fibrosis with and without associated architectural distortion, microscopic honeycombing, and airway-centered fibrosis.

### Exhaled breath analysis–electronic nose (eNose) technology

“Breathomics,” a field of study involving the analysis of particles and molecules in exhaled breath, has also garnered significant interest in the last few years as an attractive potential ILD diagnostic tool since capturing exhaled breath is both simple and non-invasive (101).

Components of exhaled breath may be analyzed using real-time quantification of small volatile compounds like nitric oxide using chemiluminescence, electrochemical or laser analyzers (102). Alternatively, exhaled breath condensate can be obtained through cooling of exhalate in a collection device for subsequent laboratory analysis. Exhaled breath condensate contains water vapor plus small amounts of both volatile and non-volatile molecules arising from the alveoli and airways. Liquid chromatography-mass spectrometry or specific enzyme immunoassays can be performed for measurement of larger, non-volatile molecules; or analysis of volatile organic compounds (VOCs) can be performed using gas chromatography–mass spectrometry (GC–MS), a high-throughput technique which separates, then identifies and quantifies molecules in complex mixtures of compounds (102).

The thousands of VOCs detectable in exhaled breath (103) are either produced endogenously as by-products of cellular metabolism or may also reflect exposure to various exogenous compounds. For example, isoprene and acetone are commonly detectable endogenously produced VOCs; whereas acetonitrile is not produced endogenously but is found in the breath of cigarette smokers (103). Altered cellular metabolism in various disease states is supported by studies of VOCs in COPD (104), lung cancer (105, 106) and other respiratory illnesses (102). Significantly elevated levels of specific amino acids and other organic compounds have been demonstrated in the exhaled breath of IPF patients when compared with healthy controls (107–109). GC–MS analysis of exhaled breath samples obtained from ILD patients and healthy controls has also been able to distinguish between IPF and connective tissue disease-associated ILD (CTD-ILD) based on discriminating VOCs (110). Importantly, in this study, VOC profiles were also associated with measures of disease severity, including total lung capacity and six-minute walk distance (110).

Electronic nose (“eNose”) technology has been developed as a novel method of exhaled breath analysis. eNoses are devices containing multiple cross-reactive gas sensors, each with partial specificity to various molecules. Upon exposure to specific VOCs, an electronic response is generated from each sensor; and a unique pattern of sensor responses is produced for the individual whose breath is being tested, known as the “breathprint” (101, 111). A preliminary eNose ILD study of 31 sarcoidosis and 25 healthy controls previously demonstrated the breathprint of patients with untreated sarcoidosis to be distinct from healthy control individuals (112). Similarly, electronic nose analysis of exhaled breath-derived VOC profiles has been shown to distinguish between IPF patients and healthy controls, and to inversely correlate with bronchoalveolar lavage fluid total cell count in a small study of 32 IPF patients, 33 COPD patients and 36 healthy controls recruited from 2 centers (113). A single center analysis of the breathprint of 174 ILD patients, 23 COPD patients and 33 healthy controls demonstrated the VOC signature of ILD patients as measured using an eNose device to be distinguishable from those of healthy controls and patients with COPD. However, the specificity of the eNose for distinguishing between different ILD subgroups was poor (114).

A larger single center, cross-sectional study undertaken in the Netherlands demonstrated that an eNose device, the SpiroNose, was able to differentiate between ILD patients and healthy control subjects based on machine learning algorithmic analysis of their breathprint (101). Additionally, these pattern recognition algorithms were able to distinguish between IPF and non-IPF ILDs, with an area under the curve (AUC) of 0.91(95% CI 0.85–0.96) in the training set and an AUC of 0.87 (95% CI 0.77–0.96) in the validation set. Furthermore, the model directly compared individual diagnoses’ breathprints and was consistently able to distinguish between different ILD subgroups including IPF, chronic hypersensitivity pneumonitis, CTD-ILD, idiopathic non-specific interstitial pneumonia, interstitial pneumonia with autoimmune features and sarcoidosis; with AUCs ranging between 0.85–0.99 (101).

Another cross-sectional study performed in the Netherlands demonstrated eNose technology to be able to reliably differentiate sarcoidosis from control participants, and from other subgroups of ILD, including chronic hypersensitivity pneumonitis (115). Importantly, preliminary studies suggest that exhaled breath analysis using an eNose may also predict treatment response. A recent analysis of 42 treatment-naïve ILD patients, showed that patients who responded to both immunosuppressive and antifibrotic therapies (defined as FVC improvement of ≥5% after 1–3 months, or FVC decline of ≤2.5%, respectively) had distinguishable exhaled VOC profiles, compared with patients who did not respond to treatment (116).

Unlike GC–MS, eNose analysis of exhaled breath VOCs has been performed in real-time, by using cloud-connected collection devices connected to validated online analysis platforms (101) and has therefore been proposed as a novel point-of-care diagnostic tool that could readily be incorporated into clinical practice (111). Importantly, breath biomarkers such as FeNO (fractional exhaled nitric oxide) can potentially be obtained and analyzed remotely; and were thus considered a novel approach for diagnosis and monitoring of chronic lung diseases during the SARS-CoV-2 pandemic (117). It is clear however that their potential extends beyond the current pandemic and that the use of eNose technology could contribute meaningfully to ILD diagnosis and patient self-management in the future, should their use be validated in larger studies.

#### Key points

1. eNoses are a novel technology capable of analyzing volatile compounds present in exhaled breath via cross-reactive gas sensors.
2. Cross-sectional studies have demonstrated eNose exhaled breath analysis to consistently be able to distinguish between ILD patients and healthy controls; and to differentiate ILD subtypes including IPF, chronic hypersensitivity pneumonitis, sarcoidosis, NSIP and CTD-ILD.

### Acoustic signatures and ultrasonography

Digital auscultation represents a novel tool to measure and digitally record lung sounds, a well-established clinical sign of ILD (118–121). Preliminary studies have evaluated multichannel lung sound analysis, using microphones placed on the anterior and posterior chest to acquire recording of lung sounds. The technique can distinguish the bibasal fine, “velcro-like” crackles associated with IPF from lung crackles due to other causes such as congestive cardiac failure (122). Clinician assessment in a tertiary ILD center with a traditional stethoscope has been shown to predict the presence of fibrotic ILD and UIP pattern as seen on HRCT, yet has clear limitations including inter-observer variability, particularly outside of this setting (123).

More recently, feasibility studies of digital lung sound recordings obtained using electronic stethoscopes have confirmed “velcro-like” crackles to be predictive of fibrotic ILD on HRCT, particularly UIP pattern (OR 19.8) and to positively correlate with specific radiologic features including reticulation, honeycombing and traction bronchiectasis (119, 120).

A 2019 pilot study of 19 IPF patients analyzing digital lung sound recordings at seven timepoints over 12 months identified a set of 19 acoustic features able to distinguish IPF patients from healthy subjects. These included features such as number of crackles, crackle onset timing and frequency range in hertz (120). Serial analysis of the digital sound recordings showed individual acoustic signatures to change over time and to correlate with markers of disease severity and progression, including visual scores of ILD extent on CT and volumetric analysis using Computer-Aided Lung Informatics for Pathology Evaluation and Rating (CALIPER) software (120). Additionally, a score integrating the acoustic signals and composite physiologic index (CPI), a well-established prognostic model in IPF (124), was better able to predict the extent of fibrosis on HRCT than the CPI alone.

Another Mexican study investigated the use of a mobile health application enabling clinicians to record and analyze respiratory sounds using a smartphone and acoustic sensors (121). Although this was only a small feasibility study, and further research into this technology is required, the prospect of this diagnostic tool is exciting since it is non-invasive and broadly accessible, potentially improving remote evaluation of ILD patients distant from tertiary centers.

There is also increasing interest in the use of thoracic ultrasound for diagnosis and monitoring of interstitial lung diseases. Currently, its use as a diagnostic tool is limited since features on ultrasound such as “B-lines,” although very sensitive, ultrasonographic signs of interlobular septal thickening in ILD, are not a specific finding, and may be seen in many other conditions including cardiac failure, atelectasis, pneumonia, and other diagnoses (125, 126). The potential for ultrasound however, as a screening tool is compelling, particularly in patients with established risk factors for ILD such as connective tissue disease (126), and for monitoring disease progression (127). A recent study evaluating lung ultrasound findings in 24 ILD patients over 12 months found that the lung ultrasound (LUS) score, the sum of B-lines counted in each intercostal space, using a standard 56-lung intercostal space LUS protocol, to correlate with HRCT Warrick score, a score of extent of radiologic fibrosis (127).

#### Key points

1. Digital analysis of lung sounds and thoracic ultrasonography might be able to detect early features of ILD, and also represent simple, non-invasive methods for monitoring of disease extent.

## Future directions

There is a currently unmet need for improved diagnostic biomarkers and tests in ILD. While there has been great progress in this field, the next steps globally are to integrate such tools into clinical care. Some biomarkers, including MMP-7 or integrated scores, genetic testing, and even some radiological AI tools are prime for clinical integration. Others, including digital auscultation and eNose technology are promising as potential screening tests for early detection of ILD in primary care and other non-expert settings, as well as offering opportunities for remote assessment and monitoring. eNose technology, EB-OCT and deep-learning based radiologic assessment are particularly attractive diagnostic tests since they do not require invasive tissue sampling. While genomic testing and deep learning-based histopathological assessment currently do require tissue sampling, this research continues to further our knowledge of the pathobiological mechanisms underlying the development and progression of fibrotic lung diseases.

The future of ILD diagnostics is promising. Feasibly, machine learning algorithms may be trained to generate virtual biopsies from radiologic or EB-OCT data, negating the need for invasive investigations altogether. Liquid biopsies from blood samples may also play a role in elucidating key transcriptomic signatures for precision disease profiling and therapeutic strategies. Regardless, further research is needed to develop and externally validate novel diagnostic techniques to improve access to timely and accurate diagnosis for ILD patients.

## Author contributions

LG wrote the manuscript, with input from LT and TC. All authors reviewed the manuscript and agreed with regard to the contents.

## Funding

This research was supported by a Lung Foundation Australia scholarship, with matched funding provided by the University of Sydney (Lung Foundation Australia/Diana Cox Idiopathic Pulmonary Fibrosis PhD Scholarship 2019).

## Conflict of interest

LG has received travel and conference support from Boehringer Ingelheim. LT has provided paid consultancy for Erbe Elektromedezin and Boehringer Ingelheim. TC has received grant support, consultancy fees, and speaking honoraria from Boehringer Ingelheim and Hoffman-La Roche, consultancy fees from Bristol Myers Squibb; grant support from Biogen, and provides consultancy for DevPro and Ad Alta.

## Publisher’s note

All claims expressed in this article are solely those of the authors and do not necessarily represent those of their affiliated organizations, or those of the publisher, the editors and the reviewers. Any product that may be evaluated in this article, or claim that may be made by its manufacturer, is not guaranteed or endorsed by the publisher.
